# Supplementary material for: The EEG signature of sensory evidence accumulation during decision formation closely tracks subjective perceptual experience
Source: Sci Rep. 2019 Mar 20;9:4949. doi: 10.1038/s41598-019-41024-4 (PMC6426990; doi:10.1038/s41598-019-41024-4)
Supplement: Supplementary file 1 — Supplementary Info [file 41598_2019_41024_MOESM1_ESM.pdf]

The EEG signature of sensory evidence accumulation during decision formation closely tracks subjective perceptual experience

Chiara F. Tagliabue[1,2*†], Domenica Veniero[3†], Christopher S. Y. Benwell[4], Roberto Cecere[3], Silvia Savazzi[2], Gregor Thut[3]

[1]CIMeC – Center for Mind/Brain Sciences, University of Trento, Rovereto, Italy

[2]Perception and Awareness (PandA) Laboratory, Department of Neuroscience, Biomedicine and Movement Sciences, University of Verona, Verona, Italy

[3]Centre for Cognitive Neuroimaging, Institute of Neuroscience and Psychology, University of Glasgow, Glasgow, UK

[4]Psychology, School of Social Sciences, University of Dundee, Dundee, UK

[†]These authors contributed equally to this study

***Corresponding author:**

Chiara F. Tagliabue

CIMeC – Center for Mind/Brain Sciences, University of Trento

Corso Bettini 31, 38068, Rovereto, Italy

chiara.tagliabue@unitn.it

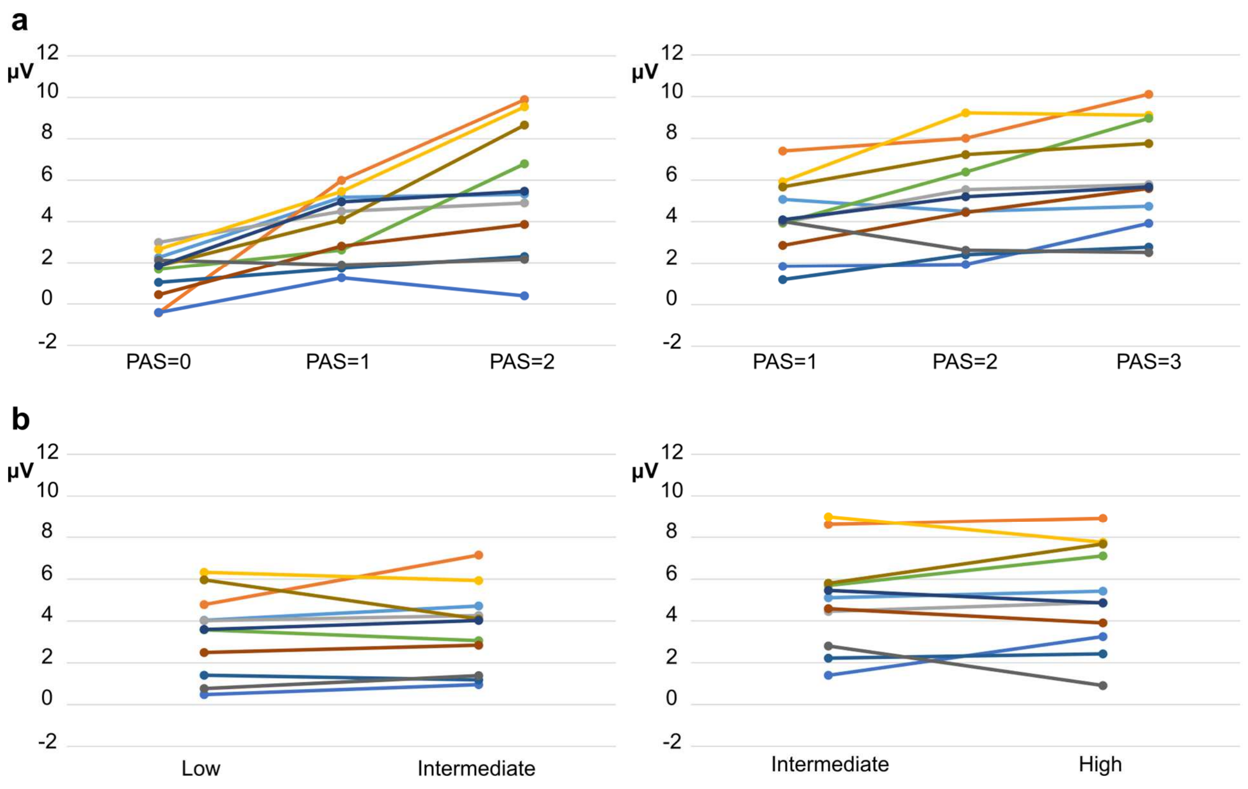

Supplementary Figure 1. ERP Data. Single participant data of the mean amplitude for electrode Pz in a 300-700ms time window for each comparison of **(a)** level of subjective evidence (when controlled for stimulus intensity) and **(b)** level of stimulus intensity (when controlled for PAS ratings). The data reveal high consistency across participants for ERP-modulations across PAS levels, and its absence for ERP modulations across intensity levels.
